# Potent Antiviral Activities of the Direct-Acting Antivirals ABT-493 and ABT-530 with Three-Day Monotherapy for Hepatitis C Virus Genotype 1 Infection

**DOI:** 10.1128/AAC.02264-15

**Published:** 2016-02-26

**Authors:** Eric J. Lawitz, William D. O'Riordan, Armen Asatryan, Bradley L. Freilich, Terry D. Box, J. Scott Overcash, Sandra Lovell, Teresa I. Ng, Wei Liu, Andrew Campbell, Chih-Wei Lin, Betty Yao, Jens Kort

**Affiliations:** a Texas Liver Institute, University of Texas Health Science Center, San Antonio, Texas, USA; b eStudySite, San Diego, California, USA; c AbbVie Inc., North Chicago, Illinois, USA; d Kansas City Gastroenterology & Hepatology, Kansas City, Missouri, USA; e Clinical Research Centers of America, Murray, Utah, USA

## Abstract

ABT-493 is a hepatitis C virus (HCV) nonstructural (NS) protein 3/4A protease inhibitor, and ABT-530 is an HCV NS5A inhibitor. These direct-acting antivirals (DAAs) demonstrated potent antiviral activity against major HCV genotypes and high barriers to resistance *in vitro*. In this open-label dose-ranging trial, antiviral activity and safety were assessed during 3 days of monotherapy with ABT-493 or ABT-530 in treatment-naive adults with HCV genotype 1 infection, with or without compensated cirrhosis. The presence of baseline resistance-associated variants (RAVs) was also evaluated. The mean maximal decreases in HCV RNA levels from baseline were approximately 4 log_10_ IU/ml for all ABT-493 doses ranging from 100 mg to 700 mg and for ABT-530 doses of ≥40 mg. There were no meaningful differences in viral load declines for patients with versus without compensated cirrhosis. Twenty-four (50%) of the baseline samples from patients treated with ABT-493 had RAVs to NS3/4A protease inhibitors. Among 40 patients treated with ABT-530, 6 (15%) carried baseline RAVs to NS5A inhibitors. Viral load declines in patients with single baseline NS5A RAVs were similar to those in patients without RAVs. One patient harbored baseline RAVs at 3 NS5A positions and appeared to have a slightly less robust viral load decline on day 3 of monotherapy. No serious or grade 3 (severe) or higher adverse events and no clinically relevant laboratory abnormalities were observed with either compound. ABT-493 and ABT-530 demonstrated potent antiviral activity and acceptable safety during 3-day monotherapy in patients with HCV genotype 1 infection, with or without compensated cirrhosis. Based on these results, phase II studies assessing the combination of these DAAs for the treatment of chronic HCV infection in patients with or without compensated cirrhosis have been initiated. (This study has been registered at ClinicalTrials.gov under registration no. NCT01995071.)

## INTRODUCTION

Hepatitis C virus (HCV) infection is a global health problem, with an estimated 184 million individuals infected globally (1). Of the 6 major HCV genotypes, genotype 1 (GT1) is the most prevalent worldwide, including in the United States (1). Up to 40% of patients with chronic HCV infection develop cirrhosis (2). The incidence of hepatocellular carcinoma among patients with HCV infections is estimated to range from 1% to 5% (2). Death related to the complications of cirrhosis may occur at an incidence of approximately 4% per year, and patients diagnosed with hepatocellular carcinoma have a 33% probability of death during the first year after its diagnosis (2). Successful eradication of HCV infection has been shown to significantly reduce the risk of disease progression and related deaths, as well as the development of hepatocellular carcinoma (3, 4).

Interferon-free therapies with direct-acting antiviral (DAA) combinations that are currently available on the market or are in development have improved sustained virological response rates, compared with interferon-containing regimens (5); however, several limitations remain. Currently approved DAA regimens do not demonstrate comparably high potency and efficacy for all HCV genotypes and subtypes or in all HCV patient populations, including patients with cirrhosis and/or previous treatment failures. Some regimens may be associated with drug interactions that limit their utility for certain patient subgroups, and some regimens include the use of ribavirin, which can pose safety concerns such as anemia and teratogenicity. In addition, treatment with DAAs may lead to the development of DAA-resistant viral variants in patients experiencing virologic failures, which may limit future treatment options for those patients. Thus, despite significant recent progress in the development of curative therapies for chronic HCV infection, there is a need for continuing research into potent anti-HCV therapies. Desired features of such future regimens would include pangenotypic antiviral activity, including activity against common resistance-associated variants (RAVs), ribavirin-free protocols, improved drug-drug interaction profiles, the convenience of once-daily (QD) dosing with few pills, and shorter treatment durations.

ABT-493 is an HCV nonstructural (NS) protein 3/4A protease inhibitor that was identified jointly by AbbVie and Enanta, and ABT-530 is an NS5A inhibitor that was identified by AbbVie. *In vitro*, the individual compounds demonstrated potent antiviral activity against all major HCV genotypes, with high barriers to resistance, and both maintained potent antiviral activity against common HCV single-position variants that confer resistance to other NS3/4A protease or NS5A inhibitors (6–8). When tested in HCV GT1b Con-1 replicon cells, the combination of ABT-493 and ABT-530 demonstrated activities that were additive to synergistic, depending on the concentrations tested (7), as assessed using the model described by Prichard and Shipman (9). Based on these characteristics, ABT-493 and ABT-530 have been designated next-generation DAAs and are being advanced for further pharmaceutical development by AbbVie.

This study assessed the antiviral activity and safety of ABT-493 and ABT-530 in treatment-naive adults with HCV GT1 infection, with or without compensated cirrhosis, during 3 days of monotherapy with each compound. The presence of baseline RAVs was also assessed.

## MATERIALS AND METHODS

### Patients and study design.

Eligible patients were adults between 18 and 70 years of age (inclusive) with chronic HCV GT1 infection who had not previously received antiviral treatment for HCV. Patients with body mass index values between 18 kg/m^2^ and 38 kg/m^2^ and plasma HCV RNA levels of >10,000 IU/ml at screening were included. Patients with or without compensated cirrhosis were eligible; furthermore, patients with cirrhosis were eligible if they had Child-Pugh scores of ≤6 and serum alpha-fetoprotein levels of ≤100 ng/ml at screening, no current or past clinical evidence of Child-Pugh class B or C disease, and no clinical history of liver decompensation. The absence of cirrhosis was documented based on one of the following: liver biopsy (e.g., Metavir score of ≤3 or Ishak score of ≤4); FibroTest score at screening of ≤0.72 and aspartate aminotransferase/platelet ratio index of ≤2; or screening FibroScan result of <12.5 kPa. The presence of cirrhosis was documented by one of the following: histological diagnosis on liver biopsy (e.g., Metavir score of >3 or Ishak score of >4) prior to or during screening; screening FibroTest score of ≥0.73 and aspartate aminotransferase/platelet ratio index of >2; or FibroScan result of ≥14.6 kPa within 6 months before or during screening. Patients were excluded if they had, at screening, evidence of hepatocellular carcinoma, positive test results for hepatitis B virus or human immunodeficiency virus, or the presence of a HCV genotype other than GT1.

**FIG 1:**
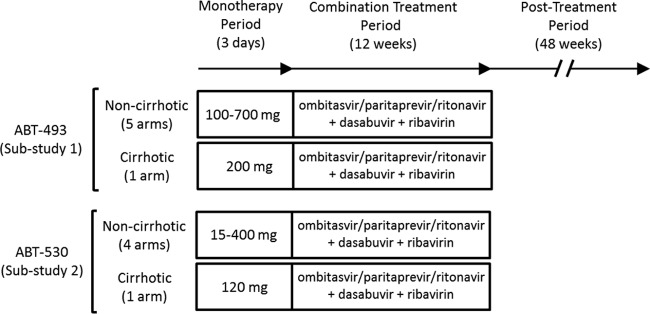
Study design. The study consisted of a 3-day monotherapy period, a 12-week combination treatment period, and a 48-week posttreatment period. Data reported here are from the 3-day monotherapy period. During the 3-day monotherapy period, doses administered in the treatment arms for patients without cirrhosis were 100, 200, 300, 400, and 700 mg (ABT-493) and 15, 40, 120, and 400 mg (ABT-530). The treatment regimen administered during the 12-week combination treatment period consisted of ombitasvir, paritaprevir, and ritonavir (25, 150, and 100 mg, respectively) administered once daily, dasabuvir (250 mg) administered twice daily, and weight-based ribavirin (total daily dose of 1,000 or 1,200 mg, divided in two).

This phase II, open-label, dose-ranging trial (ClinicalTrials registration no. NCT01995071) included 2 parallel substudies, with antiviral activity, safety, and baseline viral resistance profiles being evaluated for multiple dose levels of ABT-493 (substudy 1) or ABT-530 (substudy 2). Both compounds were administered orally QD for 3 days (Fig. 1). Each substudy included multiple dosing arms for patients without cirrhosis (total of 9 arms, i.e., 5 arms in substudy 1 and 4 arms in substudy 2) and 1 dosing arm per substudy for patients with compensated cirrhosis (total of 2 arms). Randomization occurred in a 1:1:1 ratio for the initial 3 arms in each substudy. The initial doses of ABT-493 in substudy 1 were 100 mg, 400 mg, and 700 mg, and the initial doses of ABT-530 in substudy 2 were 15 mg, 120 mg, and 400 mg. Upon assessment of available safety, HCV plasma viral load, and pharmacokinetic data from the initial 3 arms in each substudy, enrollment commenced in the subsequent arms. In substudy 1, patients were assigned to 3 additional arms, i.e., 2 for patients without cirrhosis (doses of 200 mg and 300 mg) and 1 for patients with cirrhosis (200 mg). In substudy 2, there were 2 additional arms, i.e., 1 for patients without cirrhosis (40 mg) and 1 for patients with cirrhosis (120 mg). On study day 4, after completion of 3 days of monotherapy, patients in all dosing arms were treated with the interferon-free regimen of ombitasvir, paritaprevir, and ritonavir (25, 150, and 100 mg, respectively, QD), dasabuvir (250 mg twice daily [BID]), and weight-based ribavirin (total daily dose of 1,000 or 1,200 mg, given BID) for 12 weeks. All patients who received at least one dose of any study drug were monitored for 48 weeks during the posttreatment observation period to monitor safety, HCV RNA levels, and the emergence and persistence of viral variants.

The study was designed by the study investigators and sponsor according to good clinical practice guidelines, the Declaration of Helsinki, and applicable regulations, with institutional review board approval at all study sites. All patients provided written informed consent. The sponsor conducted the data analyses. The investigators had full access to data for review and comment. The first draft of the manuscript was written by medical writers employed by the sponsor. All authors participated in manuscript development and made the final decision regarding journal submission. We confirm that the results presented are accurate and that the study was conducted and reported according to the protocol.

### Antiviral activity assessments.

The primary efficacy endpoint was the maximal decrease in HCV plasma RNA levels from baseline (log_10_ international units per milliliter) with ABT-493 or ABT-530 monotherapy for 3 days. The mean changes in HCV plasma RNA levels from baseline over time were also assessed. Plasma samples were collected on each day of the monotherapy period, immediately before dosing, 4, 8, and 12 h postdosing on day 1, 6 and 12 h postdosing on days 2 and 3, and immediately prior to the morning dose of combination treatment on day 4. Plasma HCV RNA levels were measured using the COBAS TaqMan real-time reverse transcriptase PCR assay with the High Pure System version 2.0 (Roche Molecular Diagnostics, Pleasanton, CA). The lower limit of detection was 15 IU/ml, and the lower limit of quantitation was 25 IU/ml (10).

### HCV NS3/4A and NS5A sequence analyses of baseline samples.

A plasma sample was collected from each patient on day 1, before dosing of study drugs. The relevant target, i.e., full-length NS3/4A or NS5A, was amplified from this baseline sample and analyzed by population sequencing (11, 12). Included in the analysis were clinically relevant variants at the following amino acid positions that confer resistance to one or more members of the HCV protease or NS5A inhibitor class (13): NS3 positions 36, 43, 54, 55, 56, 80, 122, 155, 156, 168, and 170 and NS5A positions 28, 29, 30, 31, 32, 58, 92, and 93.

### Safety variables.

Patients were confined during the 3 days of monotherapy, to allow study activities to be conducted during this period. Adverse events were monitored, vital signs were recorded, and laboratory tests were assessed on each of the 3 days during the monotherapy period, to evaluate the safety of ABT-493 and ABT-530, and at each subsequent clinic visit, to evaluate the safety of combination therapy.

### Statistical analyses.

The primary efficacy analysis included all patients who received at least one dose of ABT-493 or ABT-530 and had a baseline measurement and at least 1 postbaseline measurement of HCV RNA levels during the monotherapy period. The maximal decreases in HCV RNA levels were compared between doses within each substudy, such that 100 mg ABT-493 was compared to each of the higher ABT-493 doses and 15 mg ABT-530 was compared to each of the higher ABT-530 doses. These comparisons were made using an analysis of covariance, with the baseline HCV RNA level as a covariate and dose, HCV genotype subtype (1a or non-1a), and presence or absence of cirrhosis as factors. Safety and demographic analyses included all patients who received at least one dose of ABT-493 or ABT-530. SAS software version 9.3 (SAS Institute, Inc., Cary, NC) was used for all statistical summaries and analyses.

## RESULTS

### Patients.

Patients were screened at 13 centers in the United States, between November 2013 and April 2014. A total of 49 patients were enrolled in substudy 1 and received at least 1 dose of ABT-493; 48 patients completed 3-day monotherapy. In substudy 2, 40 patients received at least one dose of ABT-530, and all 40 completed 3-day monotherapy. Baseline demographics and disease characteristics were similar between dosing arms (Table 1). The majority of patients were male and white. All patients were infected with HCV GT1, with 84% (75/89 patients) being infected with GT1a, and the majority of patients had the interleukin 28B (IL-28B) non-CC genotype (74% [66/89 patients]).

**TABLE 1:** Patient demographics and baseline characteristics

Parameter^*a*^	Substudy 1 (ABT-493)						Substudy 2 (ABT-530)				
	100 mg (*n* = 8)	200 mg (*n* = 8)	200 mg, cirrhosis (*n* = 8)	300 mg (*n* = 8)	400 mg (*n* = 8)	700 mg (*n* = 9)	15 mg (*n* = 8)	40 mg (*n* = 8)	120 mg (*n* = 8)	120 mg, cirrhosis (*n* = 8)	400 mg (*n* = 8)
Male (*n* [%])	5 (62.5)	5 (62.5)	7 (87.5)	4 (50.0)	6 (75.0)	9 (100)	5 (62.5)	6 (75.0)	8 (100)	5 (62.5)	6 (75.0)
White (*n* [%])	8 (100)	7 (87.5)	7 (87.5)	6 (75.0)	8 (100)	9 (100)	6 (75.0)	8 (100)	8 (100)	6 (75.0)	8 (100)
Hispanic/Latino (*n* [%])	3 (37.5)	1 (12.5)	1 (12.5)	5 (62.5)	3 (37.5)	2 (22.2)	0	4 (50.0)	2 (25.0)	0	2 (25.0)
Age (mean ± SD) (yr)	53.8 ± 5.20	52.6 ± 6.37	58.6 ± 6.14	54.6 ± 5.15	55.8 ± 9.53	50.3 ± 9.04	53.9 ± 9.34	60.5 ± 5.61	50.6 ± 12.60	55.3 ± 9.63	49.4 ± 15.31
Weight (mean ± SD) (kg)	74.0 ± 8.39	80.6 ± 16.07	88.0 ± 12.82	85.0 ± 11.00	90.0 ± 15.21	80.7 ± 13.73	84.5 ± 15.57	82.6 ± 13.55	81.7 ± 11.53	80.9 ± 15.19	92.1 ± 25.57
HCV genotype/subtype (*n* [%])											
1a	7 (87.5)	7 (87.5)	5 (62.5)	8 (100)	6 (75.0)	9 (100)	6 (75.0)	6 (75.0)	7 (87.5)	6 (75.0)	8 (100)
1b	1 (12.5)	1 (12.5)	3 (37.5)	0	2 (25.0)	0	2 (25.0)	2 (25.0)	1 (12.5)	2 (25.0)	0
IL-28B genotype (*n* [%])											
CC	1 (12.5)	3 (37.5)	1 (12.5)	0	0	6 (66.7)	3 (37.5)	1 (12.5)	2 (25.0)	3 (37.5)	3 (37.5)
CT	5 (62.5)	5 (62.5)	6 (75.0)	7 (87.5)	7 (87.5)	2 (22.2)	2 (25.0)	6 (75.0)	4 (50.0)	5 (62.5)	3 (37.5)
TT	2 (25.0)	0	1 (12.5)	1 (12.5)	1 (12.5)	1 (11.1)	3 (37.5)	1 (12.5)	2 (25.0)	0	2 (25.0)
Baseline HCV RNA level (mean ± SD) (log_10_ IU/ml)	6.6 ± 0.81	6.8 ± 0.48	6.4 ± 0.32	6.3 ± 1.66	6.9 ± 0.25	6.9 ± 0.54	6.8 ± 0.41	6.7 ± 0.53	6.8 ± 0.47	7.1 ± 0.43	6.4 ± 0.84

a SD, standard deviation; HCV, hepatitis C virus.

### Antiviral activity.

**FIG 2:**
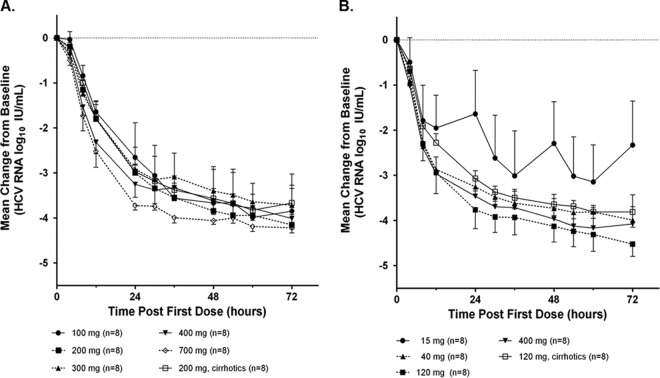
Mean changes in HCV RNA levels from baseline during monotherapy with ABT-493 or ABT-530. Data are presented as the mean changes in HCV RNA levels from baseline 0 to 72 h after the first dose for each ABT-493 (A) or ABT-530 (B) dose studied. Standard deviations are shown as error bars.

In substudy 1, the mean maximal decreases in HCV plasma RNA levels from baseline at the end of the 3 days of monotherapy ranged from 3.8 to 4.3 log_10_ IU/ml (Table 2). The maximal decrease observed with the lowest ABT-493 dose of 100 mg was not significantly different from those observed with the higher doses. There was a numerical but not statistical difference between levels for the patients with versus without cirrhosis who received the 200-mg dose of ABT-493 (levels of 3.9 versus 4.2 log_10_ IU/ml). There was no apparent relationship between doses and virologic responses (Fig. 2A).

**TABLE 2:** Summary of mean maximal decreases in HCV RNA levels from baseline

Parameter	Maximal decrease in HCV RNA level (log_10_ IU/ml)										
	Substudy 1 (ABT-493)						Substudy 2 (ABT-530)				
	100 mg (*n* = 8)	200 mg (*n* = 8)	200 mg, cirrhosis (*n* = 8)	300 mg (*n* = 8)	400 mg (*n* = 8)	700 mg (*n* = 8)	15 mg (*n* = 8)	40 mg (*n* = 8)	120 mg (*n* = 8)	120 mg, cirrhosis (*n* = 8)	400 mg (*n* = 8)
Mean	4.1	4.2	3.9	3.8	4.0	4.3	3.4	4.1	4.5	3.9	4.3
Standard deviation	0.47	0.64	0.45	1.21	0.66	0.27	0.77	0.45	0.27	0.17	0.49
Greatest change	4.5	4.9	4.5	4.8	4.7	4.7	4.3	4.6	4.9	4.2	5.0
Least change	3.2	3.3	3.2	0.9	2.6	3.7	1.9	3.2	4.0	3.7	3.6

In substudy 2, the mean maximal decreases in HCV RNA levels from baseline at the end of the 3 days of monotherapy ranged from 3.4 to 4.5 log_10_ IU/ml (Table 2) and were significantly greater for doses of 40, 120, and 400 mg ABT-530 versus 15 mg. There was no meaningful difference between levels for the patients with versus without cirrhosis who received 120 mg ABT-530 (levels of 3.9 versus 4.5 log_10_ IU/ml). Virologic responses over time in the 15-mg dose group were lower than those in the higher-dose groups (Fig. 2B). For doses of ≥40 mg, similar declines in viral load over the 3 days of monotherapy were observed, with no apparent dose-response relationship.

### HCV NS3/4A and NS5A sequence analyses at baseline.

**FIG 3:**
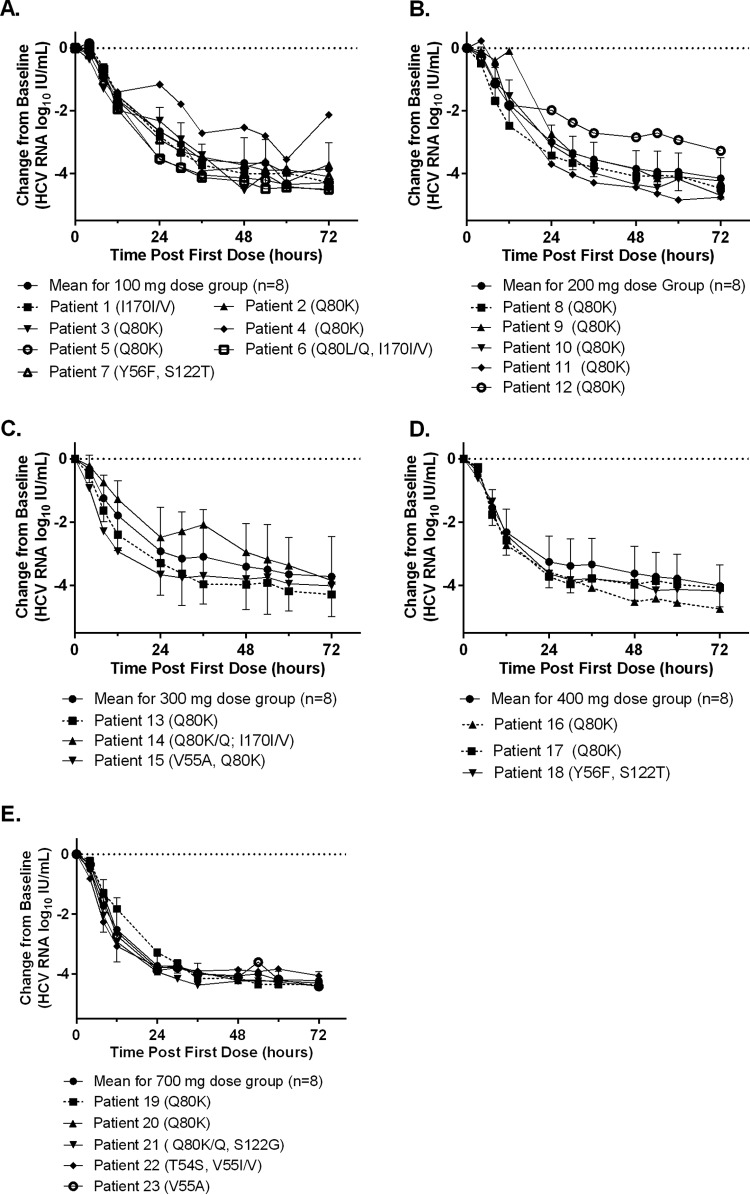
Changes in HCV RNA levels from baseline in patients with baseline NS3 RAVs who received ABT-493. Data are presented as the changes in HCV RNA levels from baseline 0 to 72 h after the first dose for each patient with baseline NS3 RAVs, compared to the mean decline curves for all patients in the corresponding ABT-493 dose groups. (A) 100 mg. (B) 200 mg. (C) 300 mg. (D) 400 mg. (E) 700 mg. Standard deviations within each dose group are shown as error bars. RAVs are listed in parentheses, and a mixture of amino acids detected at a position is denoted by a slash. One patient who had a baseline NS3 Q80K variant was not included in the HCV RNA analysis due to noncompliance.

**FIG 4:**
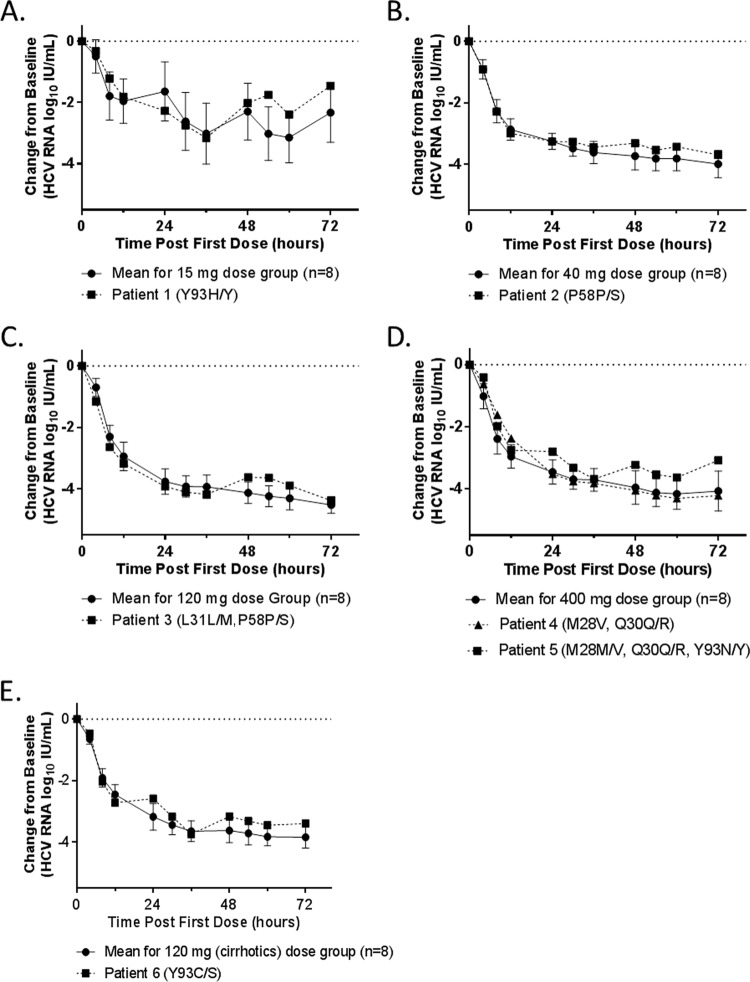
Changes in HCV RNA levels from baseline in patients with baseline NS5A RAVs who received ABT-530. Data are presented as the changes in HCV RNA levels from baseline 0 to 72 h after the first dose for each patient with baseline NS5A RAVs, compared to the mean decline curves for all patients in the corresponding ABT-530 dose groups. (A) 15 mg. (B) 40 mg. (C) 120 mg. (D) 400 mg. (E) 120 mg (with cirrhosis). Standard deviations within each dose group are shown as error bars. RAVs are listed in parentheses, and a mixture of amino acids detected at a position is denoted by a slash.

Population sequencing was conducted to detect clinically relevant variants that had been shown to be associated with resistance to HCV NS3/4A protease or NS5A inhibitors. Among patients treated with ABT-493, 24 (50%) of the baseline samples had NS3 variants associated with resistance to protease inhibitors, with 18 of them being GT1a samples containing Q80K by itself or in combination with other NS3 variants; the other NS3 variants detected were T54S, V55A/I, S122G/T, and I170V. The presence of these NS3 baseline variants did not appear to affect the viral load declines during monotherapy (Fig. 3). Two patients with Q80K at baseline (patient 4 in the 100-mg dose group and patient 12 in the 200-mg dose group) (Fig. 3) had initial viral load declines similar to those of the means of the dose groups but slightly slower viral declines after 12 h of dosing. The slower viral declines seen for these 2 patients were likely due to factors other than the presence of Q80K at baseline, as 16 of the 18 GT1a-infected patients with Q80K at baseline had viral load declines similar to the mean viral load declines in the respective dose groups. Among patients treated with ABT-530, NS5A RAVs M28V, Q30R, L31M, P58S, and/or various Y93 variants were detected in baseline samples from 6 of 40 patients (15%) (Fig. 4). Three of the 6 patients with baseline RAVs received 15 mg, 40 mg, or 120 mg ABT-530 (Fig. 4A, B, and E) and harbored RAVs as mixtures with wild-type virus at only one NS5A amino acid position. The other 3 patients with baseline RAVs received 120 mg or 400 mg ABT-530 (Fig. 4C and D) and were found to have RAVs as mixtures with wild-type virus at 2 or 3 positions. According to the protocol, all patients were randomly assigned to different arms without the information on their baseline variants; the 3 patients with multiple NS5A RAVs were assigned to the 120-mg or 400-mg ABT-530 arms by chance. The prevalences of NS3 and NS5A RAVs detected in baseline samples in this study were similar to those observed in other clinical studies (11, 14, 15). Since these results were generated by population sequencing, it was not possible to determine whether the RAVs in samples with multiple RAVs were linked. Each of those RAVs, if present as a single NS5A variant, is known to confer high levels of resistance to several NS5A inhibitors but not to ABT-530 (6, 8, 14). The individual viral load decline curves for patients with one or more baseline NS5A RAVs, with the exception of patient 5, were similar to those for patients without any NS5A RAVs, as most data points for individuals with baseline NS5A RAVs were within the standard deviation of the mean decline curve for all patients in the corresponding ABT-530 dose group (Fig. 4). Patient 5 had baseline RAVs at 3 NS5A amino acid positions (M28, Q30, and Y93) and appeared to have slightly less robust viral load decline on day 3 of monotherapy (Fig. 3C).

### Safety.

At least 1 adverse event was reported in each dose group in substudy 1 and substudy 2 (Table 3). Most adverse events were grade 1 (mild, according to NCI criteria [16]). There were no serious adverse events, adverse events of grade 3 (severe) or greater, or deaths reported during monotherapy treatment. In substudy 1, 1 patient (1/49 patients [2.0%]; 700-mg dose group) was discontinued from further dosing with ABT-493 due to the treatment-emergent adverse event of drug withdrawal syndrome during monotherapy; this adverse event was not considered by the investigators to be related to the study drug.

**TABLE 3:** Summary of treatment-emergent adverse events during monotherapy

AE^*a*^	No. (%)										
	Substudy 1 (ABT-493)						Substudy 2 (ABT-530)				
	100 mg (*n* = 8)	200 mg (*n* = 8)	200 mg, cirrhosis (*n* = 8)	300 mg (*n* = 8)	400 mg (*n* = 8)	700 mg (*n* = 9)	15 mg (*n* = 8)	40 mg (*n* = 8)	120 mg (*n* = 8)	120 mg, cirrhosis (*n* = 8)	400 mg (*n* = 8)
Any AE	3 (37.5)	6 (75.0)	6 (75.0)	2 (25.0)	3 (37.5)	2 (22.2)	3 (37.5)	1 (12.5)	2 (25.0)	3 (37.5)	1 (12.5)
Any AE with reasonable possibility of being related to DAA^*b*^	2 (25.0)	4 (50.0)	5 (62.5)	1 (12.5)	2 (25.0)	1 (11.1)	3 (37.5)	0	1 (12.5)	2 (25.0)	0
Any AE of at least grade 3	0	0	0	0	0	0	0	0	0	0	0
Any serious AE	0	0	0	0	0	0	0	0	0	0	0
Any AE leading to discontinuation of study drug	0	0	0	0	0	1 (11.1)	0	0	0	0	0

a AE, adverse event; DAA, direct-acting antiviral.

b As assessed by investigators.

Headache, diarrhea, and nausea were the commonly reported adverse events in both substudies, with headache being the most frequent. Other adverse events reported by more than 1 patient are shown in Table 4. Review of vital signs, electrocardiographic data, and laboratory data (chemistry, hematology, and urinalysis data) did not show any clinically relevant abnormalities.

**TABLE 4:** Treatment-emergent adverse events occurring in >1 patient in either substudy during monotherapy treatment period

Adverse event	No. (%)^*a*^	
	Substudy 1 (ABT-493) (*n* = 49)	Substudy 2 (ABT-530) (*n* = 40)
Headache	11 (22.4)	4 (10.0)
Diarrhea	3 (6.1)	1 (2.5)
Abdominal discomfort	3 (6.1)	0
Fatigue	3 (6.1)	0
Rash	3 (6.1)	0
Dizziness	2 (4.1)	0
Myalgia	2 (4.1)	0
Nausea	2 (4.1)	2 (5.0)
Pruritus	2 (4.1)	0
Constipation	0	2 (5.0)

a Number of treatment-emergent adverse events that occurred in patients from any dose group in either substudy.

## DISCUSSION

In this study, ABT-493 and ABT-530 each demonstrated robust antiviral activity in treatment-naive patients infected with HCV GT1. Three-day monotherapy with ABT-493 resulted in an approximately 4-log decrease in HCV plasma RNA levels across all doses studied. Three-day monotherapy with ABT-530 demonstrated an approximately 4-log decrease in HCV plasma RNA levels at doses of 40 mg or greater, with a smaller decline with the 15-mg dose.

Both antiviral agents were well tolerated during the 3 days of monotherapy in this study. Adverse events reported during monotherapy with ABT-493 or ABT-530 were predominantly mild in severity, with no observed serious adverse events or adverse events classified as grade 3 (severe) or greater. There were no clinically relevant abnormalities in vital signs, electrocardiographic findings, or laboratory data.

Patients with cirrhosis may be difficult to treat and to cure, and only one other study of short-term monotherapy with simeprevir did not exclude patients with cirrhosis (17). We included patients with compensated cirrhosis in this study to identify any potential differences in the antiviral activity and safety of ABT-493 and ABT-530 in this population. Similar viral responses and safety profiles were observed in patients with and without compensated cirrhosis when 200 mg ABT-493 and 120 mg ABT-530 were administered to these patient groups in our study.

Studies assessing 3 to 8 days of monotherapy with NS3/4A protease inhibitors (boceprevir, telaprevir, simeprevir, vedroprevir, and paritaprevir-ritonavir) in patients with chronic HCV GT1 infection reported mean or median maximal decreases in HCV RNA levels from baseline ranging from 0.8 to 4.1 log_10_ IU/ml (11, 17–23). In particular, mean or median decreases of <1 log_10_ IU/ml were seen with a 60-mg dose of vedroprevir (19–21), and decreases of 1 to 2 log_10_ IU/ml were seen with 200 to 400 mg boceprevir (22). Mean decreases of >4 log_10_ IU/ml were associated with 50 to 100 mg paritaprevir-ritonavir (11). Short-term monotherapy with newer NS3/4A protease inhibitors showed improved viral load reductions, compared with older compounds. Studies assessing 3 to 7 days of monotherapy with next-generation NS3/4A protease inhibitors (MK-5172 or GS-9857) reported mean or median maximal decreases in HCV RNA levels from baseline ranging from 3.9 to 5.4 log_10_ IU/ml (24, 25), comparable to results reported here for ABT-493 (Table 2). For most NS3/4A protease inhibitors, including ABT-493, no serious adverse events were observed during clinical studies, and most of the adverse events were deemed mild to moderate in severity (17, 18, 25). Headache was the most commonly reported adverse event, and nausea, diarrhea, and fatigue were also frequently reported by patients treated with these compounds.

For NS5A inhibitors (ledipasvir, daclatasvir, and ombitasvir), mean or median maximal decreases in HCV RNA levels from baseline ranged from 2.4 to 4.1 log_10_ IU/ml following 3 to 14 days of monotherapy in patients with chronic HCV GT1 infection (26–28). Improved viral load reductions were reported for next-generation NS5A inhibitors. Three- to 5-day monotherapy with next-generation NS5A inhibitors (GS-5816, MK-8742, or TD-6450) resulted in mean or median maximal decreases in HCV RNA levels from baseline ranging from 3.7 to 5.1 log_10_ IU/ml (29–33). A single dose of 50 to 300 mg ACH-3102, another next-generation NS5A inhibitor, resulted in mean maximal decreases in HCV RNA levels from baseline ranging from 3.7 to 3.9 log_10_ IU/ml, with levels returning to baseline at postdose day 15 (34). Data reported for these next-generation NS5A inhibitors are comparable to results reported here for ABT-530 (Table 2). NS5A inhibitors, including ABT-530, were generally well tolerated. Most adverse events were mild or moderate in severity, and headache was reported more frequently than other adverse events (26, 27, 29, 33).

In addition to the improved antiviral activity, ABT-493 and ABT-530 maintain antiviral activity against common RAVs that often negatively affect the potency of other DAAs (6–8). In this study, the prevalences of NS3 and NS5A RAVs detected in baseline samples were similar to those observed in published studies (11, 14, 15). NS3 RAVs were detected in baseline samples from 50% of patients treated with ABT-493, with Q80K being the most common RAV. The presence of baseline NS3 RAVs did not appear to affect viral load declines during ABT-493 monotherapy. Among patients receiving ABT-530, 15% had baseline NS5A variants that, if present as variants with single amino acid changes, confer notable resistance to several NS5A inhibitors, including ombitasvir, daclatasvir, and/or ledipasvir, but not to ABT-530 (8). Viral load decline curves with ABT-530 monotherapy for patients with these baseline NS5A RAVs showed little difference from those for patients without baseline NS5A RAVs, with the exception of patient 5. This patient harbored baseline RAVs at 3 NS5A positions and had a slightly smaller viral load reduction at the end of monotherapy, compared with the mean for the dose group; linkage of these 3 RAVs cannot be ruled out, based on the population sequencing performed. However, this patient achieved a sustained virological response at posttreatment week 12, following 12 weeks of treatment with the combination of ombitasvir-paritaprevir-ritonavir with dasabuvir and ribavirin (data not shown). Taken together, these monotherapy results underscore the potent antiviral activity of both ABT-493 and ABT-530 and suggest that the combination of these next-generation DAAs holds promise for more difficult-to-treat patients who harbor NS5A RAVs that are known to confer resistance to currently approved NS5A inhibitors, as well as for patients with cirrhosis. Based on the monotherapy data presented in this study and *in vitro* antiviral data for each compound, the combination of ABT-493 and ABT-530 has been advanced into phase II clinical studies with patients with genotype 1 to 6 infections, including patients with compensated cirrhosis.

## References

[1] Mohd Hanafiah K, Groeger J, Flaxman AD, Wiersma ST 2013 Global epidemiology of hepatitis C virus infection: new estimates of age-specific antibody to HCV seroprevalence. Hepatology 57:1333–1342. doi:10.1002/hep.26141. 23172780

[2] European Association for the Study of the Liver. 2014 EASL clinical practice guidelines: management of hepatitis C virus infection. J Hepatol 60:392–420. doi:10.1016/j.jhep.2013.11.003. 24331294

[3] Backus LI, Boothroyd DB, Phillips BR, Belperio P, Halloran J, Mole LA 2011 A sustained virologic response reduces risk of all-cause mortality in patients with hepatitis C. Clin Gastroenterol Hepatol 9:509–516. doi:10.1016/j.cgh.2011.03.004. 21397729

[4] Cardoso AC, Moucari R, Figueiredo-Mendes C, Ripault MP, Giuily N, Castelnau C, Boyer N, Asselah T, Martinot-Peignoux M, Maylin S, Carvalho-Filho RJ, Valla D, Bedossa P, Marcellin P 2010 Impact of peginterferon and ribavirin therapy on hepatocellular carcinoma: incidence and survival in hepatitis C patients with advanced fibrosis. J Hepatol 52:652–657. doi:10.1016/j.jhep.2009.12.028. 20346533

[5] Gilead Sciences. 2014 Sovaldi (sofosbuvir): prescribing information. Gilead Sciences, Foster City, CA.

[6] Ng T, Krishnan P, Kati W, Reisch T, Lu L, Dekhtyar T, Molla A, Collins C, Pilot-Matias T 2014 ABT-530, an HCV NS5A inhibitor with potent pangenotypic activity and high genetic barrier to resistance. Abstr 21st Annu Conf Retroviruses Opportunistic Infect, abstr 639.

[7] Ng T, Reisch T, Middleton T, McDaniel K, Kempf D, Lu L, Wang G, Jiang L, Or YS, Pilot-Matias T 2014 ABT-493, a potent HCV NS3/4A protease inhibitor with broad genotypic coverage. Abstr 21st Annu Conf Retroviruses Opportunistic Infect, abstr 636.

[8] Ng T, Pilot-Matias T, Liangjun L, Reisch T, Dekhtyar T, Krishnan P, Beyer J, Tripathi R, Pithawalla RB, Asatryan A, Campbell AL, Kort J, Collins C 2014 A next generation HCV DAA combination: potent, pangenotypic inhibitors ABT-493 and ABT-530 with high barriers to resistance. Hepatology 60:1142A.

[9] Prichard MN, Shipman C Jr 1990 A three-dimensional model to analyze drug-drug interactions. Antiviral Res 14:181–205. doi:10.1016/0166-3542(90)90001-N. 2088205

[10] Colucci G, Ferguson J, Harkleroad C, Lee S, Romo D, Soviero S, Thompson J, Velez M, Wang A, Miyahara Y, Young S, Sarrazin C 2007 Improved COBAS TaqMan hepatitis C virus test (version 2.0) for use with the High Pure system: enhanced genotype inclusivity and performance characteristics in a multisite study. J Clin Microbiol 45:3595–3600. doi:10.1128/JCM.01320-07. 17898157 PMC2168538

[11] Pilot-Matias T, Tripathi R, Cohen D, Gaultier I, Dekhtyar T, Lu L, Reisch T, Irvin M, Hopkins T, Pithawalla R, Middleton T, Ng T, McDaniel K, Or YS, Menon R, Kempf D, Molla A, Collins C 2015 In vitro and in vivo antiviral activity and resistance profile of the hepatitis C virus NS3/4A protease inhibitor ABT-450. Antimicrob Agents Chemother 59:988–997. doi:10.1128/AAC.04227-14. 25451053 PMC4335891

[12] Krishnan P, Beyer J, Mistry N, Koev G, Reisch T, DeGoey D, Kati W, Campbell A, Williams L, Xie W, Setze C, Molla A, Collins C, Pilot-Matias T 2015 In vitro and in vivo antiviral activity and resistance profile of ombitasvir, an inhibitor of hepatitis C virus NS5A. Antimicrob Agents Chemother 59:979–987. doi:10.1128/AAC.04226-14. 25451055 PMC4335823

[13] Lontok E, Harrington P, Howe A, Kieffer T, Lennerstrand J, Lenz O, McPhee F, Mo H, Parkin N, Pilot-Matias T, Miller V 2015 Hepatitis C virus drug resistance-associated substitutions: state of the art summary. Hepatology 62:1623–1632. doi:10.1002/hep.27934. 26095927

[14] Krishnan P, Tripathi R, Irvin M, Beyer J, Reisch T, Schnell G, Xie W, Zhou X, Larsen L, Kofron J, Cohen D, Podsadecki T, Pilot-Matias T, Collins C 2014 Lack of impact of baseline resistance-associated variants (RAVS) on treatment outcome in the Aviator Study with ABT-450/r, ABT-333, and ABT-267, +/− ribavirin. J Hepatol 60(Suppl 1):S498. doi:10.1016/S0168-8278(14)61390-8.

[15] Wong KA, Worth A, Martin R, Svarovskaia E, Brainard DM, Lawitz EJ, Miller MD, Mo H 2013 Characterization of hepatitis C virus resistance from a multiple-dose clinical trial of the novel NS5A inhibitor GS-5885. Antimicrob Agents Chemother 57:6333–6340. doi:10.1128/AAC.02193-12. 23877691 PMC3837913

[16] National Cancer Institute. 2009 Common terminology criteria for adverse events (CTCAE), version 4.03. National Institutes of Health, Bethesda, MD http://evs.nci.nih.gov/ftp1/CTCAE/CTCAE_4.03_2010-06-14_QuickReference_5x7.pdf.

[17] Reesink HW, Fanning GC, Farha KA, Weegink C, Van Vliet A, van't Klooster G, Lenz O, Aharchi F, Marien K, Van Remoortere P, de Kock H, Broeckaert F, Meyvisch P, Van Beirendonck E, Simmen K, Verloes R 2010 Rapid HCV-RNA decline with once daily TMC435: a phase I study in healthy volunteers and hepatitis C patients. Gastroenterology 138:913–921. doi:10.1053/j.gastro.2009.10.033. 19852962

[18] Lenz O, de Bruijne J, Vijgen L, Verbinnen T, Weegink C, Van Marck H, Vandenbroucke I, Peeters M, Simmen K, Fanning G, Verloes R, Picchio G, Reesink H 2012 Efficacy of re-treatment with TMC435 as combination therapy in hepatitis C virus-infected patients following TMC435 monotherapy. Gastroenterology 143:1176–1178.e6. doi:10.1053/j.gastro.2012.07.117. 22885330

[19] Dvory-Sobol H, Wong KA, Ku KS, Bae A, Lawitz EJ, Pang PS, Harris J, Miller MD, Mo H 2012 Characterization of resistance to the protease inhibitor GS-9451 in hepatitis C virus-infected patients. Antimicrob Agents Chemother 56:5289–5295. doi:10.1128/AAC.00780-12. 22869562 PMC3457362

[20] Lawitz EJ, Hill JM, Marbury T, Demicco MP, Delaney W, Yang J, Moorehead L, Mathias A, Mo H, McHutchison JG, Rodriguez-Torres M, Gordon SC 2013 A phase I, randomized, placebo-controlled, 3-day, ascending-dose study of GS-9451, an NS3/4A protease inhibitor, in genotype 1 hepatitis C patients. Antivir Ther 18:311–319. doi:10.3851/IMP2415. 23047118

[21] Yang J, Hongmei H, Dvory-Sobol H, Lawitz EJ, Moorehead L, Mathias A, Delaney W, McHutchinson J 2012 Antiviral activity of GS-9451, an HCV specific NS3 protease inhibitor, in a three-day, dose-ranging, monotherapy study in patients with genotype 1 hepatitis C infection. Hepatol Int 6:176.

[22] Sarrazin C, Rouzier R, Wagner F, Forestier N, Larrey D, Gupta SK, Hussain M, Shah A, Cutler D, Zhang J, Zeuzem S 2007 SCH 503034, a novel hepatitis C virus protease inhibitor, plus pegylated interferon α-2b for genotype 1 nonresponders. Gastroenterology 132:1270–1278. doi:10.1053/j.gastro.2007.01.041. 17408662

[23] Sarrazin C, Kieffer TL, Bartels D, Hanzelka B, Muh U, Welker M, Wincheringer D, Zhou Y, Chu HM, Lin C, Weegink C, Reesink H, Zeuzem S, Kwong AD 2007 Dynamic hepatitis C virus genotypic and phenotypic changes in patients treated with the protease inhibitor telaprevir. Gastroenterology 132:1767–1777. doi:10.1053/j.gastro.2007.02.037. 17484874

[24] Gentile I, Buonomo AR, Borgia F, Zappulo E, Castaldo G, Borgia G 2014 MK-5172: a second-generation protease inhibitor for the treatment of hepatitis C virus infection. Expert Opin Investig Drugs 23:719–728. doi:10.1517/13543784.2014.902049. 24666106

[25] Rodriguez-Torres M, Glass S, Hill J, Freilich B, Hassman D, Di Bisceglie A, Taylor JG, Kirby B, Yang JC, Stamm LM, Brainard DM, Kim S, Krefetz D, Smith W, Marbury T, Lawitz EJ 2015 The pan-genotypic NS3/4A protease inhibitor GS-9857 demonstrates potent antiviral activity in patients infected with HCV genotype 1, 2, 3, or 4 in a 3-day monotherapy study. J Hepatol 62(Suppl 2):S682. doi:10.1016/S0168-8278(15)31104-1.

[26] Lawitz EJ, Gruener D, Hill JM, Marbury T, Moorehead L, Mathias A, Cheng G, Link JO, Wong KA, Mo H, McHutchison JG, Brainard DM 2012 A phase 1, randomized, placebo-controlled, 3-day, dose-ranging study of GS-5885, an NS5A inhibitor, in patients with genotype 1 hepatitis C. J Hepatol 57:24–31. doi:10.1016/j.jhep.2011.12.029. 22314425

[27] Nettles RE, Gao M, Bifano M, Chung E, Persson A, Marbury TC, Goldwater R, DeMicco MP, Rodriguez-Torres M, Vutikullird A, Fuentes E, Lawitz EJ, Lopez-Talavera JC, Grasela DM 2011 Multiple ascending dose study of BMS-790052, a nonstructural protein 5A replication complex inhibitor, in patients infected with hepatitis C virus genotype 1. Hepatology 54:1956–1965. doi:10.1002/hep.24609. 21837752

[28] Lawitz E, Marbury T, Campbell A, Dumas E, Kapoor M, Pilot-Matias T, Krishnan P, Setze C, Xie W, Podsadecki T, Bernstein B, Williams L 2012 Safety and antiviral activity of ABT-267, a novel NS5A inhibitor, during 3-day monotherapy: first study in HCV genotype-1 (GT1)-infected treatment-naive subjects. J Hepatol 56(Suppl 2):S469–S470. doi:10.1016/S0168-8278(12)61198-2.

[29] Hebner C, Gontcharova V, Chodavarapu RK, Rodriguez-Torres M, Lawitz EJ, Yang C, McNally J, Link JO, Mo H 2013 Deep sequencing of HCV NS5A from a 3-day study of GS-5816 monotherapy confirms the potency of GS-5816 against pre-existing genotype 1–3 NS5A resistance-associated variants. Hepatology 58:433A–434A.

[30] Liu R, Curry S, McMonagle P, Nachbar RB, Pak I, Jumes P, Ludmerer S, Asante Appiah E, Hazuda D, Yeh WW, Howe AY 2013 Resistance analysis of genotype-1 and -3 HCV-infected patients receiving MK-8742, a HCV NS5A inhibitor with potent antiviral activity. Hepatology 58:435A.

[31] Yeh WW, Lipardi C, Jumes P, De Lepeleire IM, Van den Bulk N, Caro L, Huang X, Mangin E, Nachbar RB, Gane EJ, Popa S, Ghicavii N, Wagner FD, Butterton JR 2013 MK-8742, a HCV NS5A inhibitor with a broad spectrum of HCV genotypic activity, demonstrates potent antiviral activity in genotype-1 and -3 HCV-infected patients. Hepatology 58:438A–439A.

[32] Lawitz E, Rodriguez-Torres M, Kohler R, Amrite A, Barnes C, Pecoraro MLC, Budman J, McKinnell M, Washington CB 2015 TD-6450, a next generation once-daily NS5A inhibitor, has potent antiviral activity following a 3-day monotherapy study in genotype 1 HCV infection. J Hepatol 62(Suppl 2):S680–S681. doi:10.1016/S0168-8278(15)31101-6.

[33] Lawitz E, Freilich B, Link J, German P, Mo H, Han L, Brainard DM, McNally J, Marbury T, Rodriguez-Torres M 2015 A phase 1, randomized, dose-ranging study of GS-5816, a once-daily NS5A inhibitor, in patients with genotype 1–4 hepatitis C virus. J Viral Hepat 22:1011–1019. doi:10.1111/jvh.12435. 26183611

[34] Muir A, Hill J, Lawitz E, Marbury T, Robarge L, Robison H, Hui J, Huang M, Agarwal A, Perelson A, Deshpande M, Kocinsky H 2013 ACH-3102, a second generation NS5A inhibitor, demonstrates potent antiviral activity in patients with genotype 1a HCV infection despite the presence of baseline NS5A-resistant variants. J Hepatol 58(Suppl 1):S360. doi:10.1016/S0168-8278(13)60878-8.

